# Caudal Regression Syndrome First Diagnosed in Adulthood: A Case Report and a Review of the Literature

**DOI:** 10.3390/diagnostics14101000

**Published:** 2024-05-11

**Authors:** Intars Bulahs, Agnete Teivāne, Ardis Platkājis, Arturs Balodis

**Affiliations:** 1 Faculty of Residency, Riga Stradins University, 1007 Riga, Latvia025712@rsu.edu.lv (A.T.); 2 Institute of Diagnostic Radiology, Pauls Stradins Clinical University Hospital, 1002 Riga, Latvia; 3 Department of Neurology, Pauls Stradins Clinical University Hospital, 1002 Riga, Latvia; 4 Department of Radiology, Riga Stradins University, 1007 Riga, Latvia; ardis.platkajis@rsu.lv

**Keywords:** caudal regression syndrome, diastematomyelia, dorsal dermal sinus tract, sacral dysplasia

## Abstract

**Background:** Caudal regression syndrome (CRS) is a rare congenital malformation characterized by incomplete development of the lower spine and spinal cord. Its estimated incidence ranges from 1 to 2 per 100,000 live births, leading to a spectrum of clinical presentations. Although most cases are diagnosed during childhood, only a small number of cases have been documented in adults in the medical literature. **Case Report:** A 27-year-old woman underwent an outpatient magnetic resonance imaging (MRI) of the thoracolumbar spine due to severe lower back pain experienced for the first time. Despite congenital leg abnormalities and multiple childhood surgeries, no further investigations were conducted at that time. MRI revealed congenital anomalies consistent with CRS, including coccygeal agenesis, L5 sacralization, and spinal cord defects. The patient also had a long-standing pilonidal cyst treated conservatively, now requiring operative treatment due to an abscess. **Conclusions:** This report underscores a rare case of CRS initially misdiagnosed and mistreated over many years. It emphasizes the importance of considering less common diagnoses, especially when initial investigations yield inconclusive results. This clinical case demonstrates a highly valuable and educative radiological finding. In the literature, such cases with radiological findings in adults are still lacking.

## 1. Introduction

Caudal regression syndrome (CRS) encompasses a group of rare congenital malformations primarily affecting the lower spinal region, including the spinal cord and vertebrae. CRS is also sometimes referred to in the literature as caudal dysplasia sequence, sacral regression syndrome or sacral agenesis. The estimated incidence of CRS ranges from 1 to 2 per 100,000 live births, with an equal distribution between genders [1,2]. Several maternal risk factors have been identified that can increase the likelihood of CRS, including folate deficiency, maternal obesity, and exposure to teratogenic medications during pregnancy. Of particular significance is gestational diabetes, which represents a strong risk factor for CRS. Studies suggest that the risk of CRS is markedly elevated in pregnancies complicated by gestational diabetes, estimated to be 200 to 400 times higher compared to the general population [3,4,5,6].

Prenatal diagnosis of CRS is feasible, particularly in more severe cases; however, the majority of cases are typically identified postnatally. Mild clinical presentations may even go undetected until adulthood. According to a recent case review by Qudsieh et al., the majority of diagnoses occur during infancy, typically within the first year of life, although there are rare instances where patients are diagnosed after puberty [2]. Early diagnosis of CRS is critical for ensuring optimal clinical outcomes and enhancing the patient’s quality of life through timely medical interventions. The clinical presentation of CRS varies widely, ranging from asymptomatic cases to severe neurological deficits, and encompassing genitourinary, gastrointestinal, and musculoskeletal anomalies and symptoms. It is noteworthy that adults with CRS may either remain asymptomatic or develop symptoms later in life, such as lumbosacral pain, perineal discomfort, or enuresis attributed to tethered cord syndrome [2,7,8].

Neurologically, individuals affected by CRS may exhibit a diverse range of symptoms arising from intricate spinal and neural abnormalities. These symptoms encompass neurogenic bladder dysfunction, which can manifest as either urinary incontinence or retention, as well as bowel disturbances like constipation or fecal incontinence. Lower extremity weakness and sensory deficits in specific dermatomal distributions are also commonly observed manifestations. Moreover, tethered cord syndrome, a frequent complication associated with CRS, presents with progressive neurological symptoms that significantly impact daily functioning. This syndrome in adults often manifests as lumbosacral pain, exacerbated by activities such as walking or prolonged standing. Bladder dysfunction is another characteristic feature, involving difficulties with urination and potential urinary leakage. MRI findings associated with tethered cord include myelomeningocele, lipomyelomeningocele, diastematomyelia, dorsal dermal sinus, tumors, or any other inelastic structures (such as fibrous tissue resulting from prior surgery, infection, or hemorrhage). Surgical intervention should be considered for patients with clinical and radiological signs of tethered cord syndrome. Long-term outcomes after surgery are favorable, resolving or improving symptoms in most patients, but in 30% of cases a second or even third surgical intervention might be needed to untether the spinal cord. Additionally, lower extremity weakness can lead to challenges with mobility and motor function, while sensory loss may manifest as altered sensation or numbness in specific regions of the body delineated by dermatomes [1,9,10,11].

Musculoskeletal anomalies commonly associated with CRS encompass a spectrum of abnormalities that impact the lower spinal region and extremities. These include sacral agenesis, characterized by partial or complete absence of the sacrum, multiple vertebral anomalies resulting in spinal deformities like scoliosis, leg length discrepancies, and various foot deformities such as clubfoot. In addition to these musculoskeletal manifestations, CRS may also be linked with other congenital syndromes, further expanding its clinical spectrum. For instance, CRS can co-occur with syndromes like VACTERL (vertebral defects, anal atresia, cardiac defects, tracheoesophageal fistula, renal anomalies, limb abnormalities), OEIS (omphalocele, exstrophy of the cloaca, imperforate anus, spinal defects), and the Currarino triad (anorectal malformation, sacral bone abnormality, presacral mass). The presence of these syndromic associations can result in a wider range of clinical presentations and additional systemic manifestations beyond the musculoskeletal system [5,12,13].

Cutaneous malformations are commonly observed in pediatric patients with congenital spinal cord malformations, with a significant proportion—approximately 70%—exhibiting at least one of the high-risk cutaneous markers. These cutaneous markers serve as important indicators of underlying neurological anomalies and require careful evaluation and follow-up. Examples of these high-risk cutaneous markers include hypertrichosis (excessive hair growth), dermal sinus tract (an abnormal connection between the skin and the spinal canal), infantile hemangioma (a benign vascular tumor), atretic meningocele (a type of neural tube defect), and subcutaneous lipoma (a benign fatty tumor). The identification of these high-risk skin markers necessitates additional neurological and MRI examinations [14,15,16].

Sacral agenesis is a prominent hallmark characteristic of CRS, representing a critical aspect of the congenital spinal malformations observed in affected individuals. This condition is often classified using Pang’s classification system, which categorizes sacral agenesis into five morphological variants ranging from partial to total sacral agenesis, with varying degrees of vertebral involvement. Pang’s classification system provides a comprehensive framework for characterizing the extent and severity of sacral agenesis, aiding in clinical assessment and treatment planning. Additionally, Renshaw’s classification system is commonly employed to categorize sacral agenesis and related malformations. Renshaw’s classification closely resembles Pang’s system but may differ in specific details and criteria (see Table 1 for reference). Notably, some scholars recognize a fifth type of sacral agenesis known as sirenomelia, characterized by a severe form of caudal dysgenesis where the legs are fused together. Sirenomelia is the most severe form of CRS, mostly described in human fetuses (premature births, stillborn children) or in pediatric patients who died in the first days of life; most commonly, morbidity is associated with urinary tract (bilateral renal agenesis) and cardiac malformations. Some patients might survive up to one year if urogenital and anus surgeries are performed. Sirenomelia may exhibit various associated leg deformities, prompting further subclassification based on the specific anatomical variations observed [1,7,17,18,19,20].

In CRS, spinal cord termination is classified into two primary types: Type 1 and Type 2, each representing distinct patterns of spinal cord malformation and associated anomalies. Type 1 CRS is characterized by a shortened spine, high-lying cord termination, and pronounced visceral (internal organ) and orthopedic (bone and joint) anomalies. Conversely, Type 2 CRS is considered less severe, featuring a low-lying cord termination and milder malformations [1,9,21]. An intriguing aspect of CRS is the observed correlation between the severity of sacral anomalies and the level at which the spinal cord terminates. This correlation suggests a potential association between abnormal spinal cord development and the surrounding tissues during embryonic formation. The intricate interplay between spinal cord development and adjacent structures underscores the complex nature of congenital malformations like CRS, involving multiple developmental processes and genetic factors.

Treatment is limited and complex, requiring extensive diagnostic examinations and a multidisciplinary approach to a patient’s individual anatomical defects and needs. Most interventions are performed to prevent life threatening conditions in early childhood such as tracheoesophageal fistula, cloacal anomaly, omphalocele, bladder exstrophy, and imperforate anus. Some quality of life interventions are available, especially orthopedic treatment of spine and lower limbs, but patients with severe presentation may still have a remaining variable degree of functional deficits or deformity after treatment [1].

## 2. Case Report

**Figure 1:**
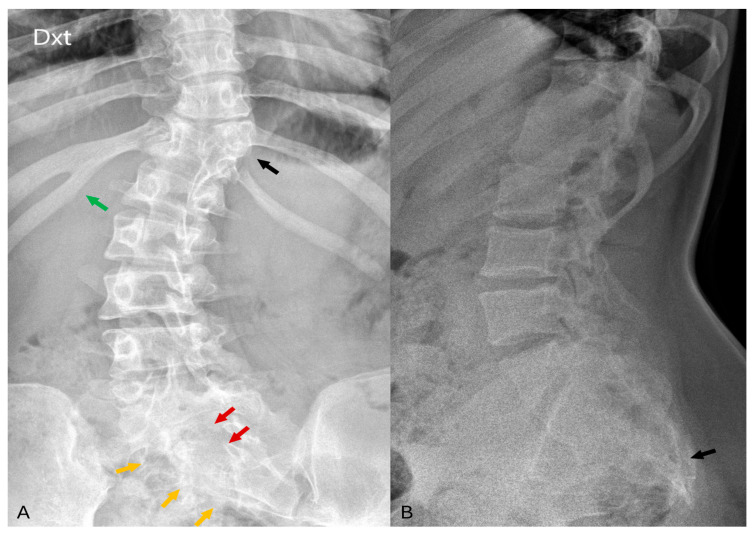
(**A**) In the AP projection of the lumbar spine radiographs, notable findings include right-sided fusion of the 11th and 12th ribs (green arrow), partial segmentation of the 11th thoracic vertebra (black arrow), and posterior arch defects observed in the sacral vertebrae (red arrows). Suspicious structural changes in the right side of the sacrum can also be visible (orange arrows). (**B**) In the LL projection of the lumbar spine, visualization reveals a deformed and shortened sacrum as well as coccyx agenesis (black arrow).

A 27-year-old woman presented with a one-month history of severe lower back pain radiating to the thoracic region, accompanied by a sub-febrile fever. This constellation of symptoms prompted an outpatient MRI of the thoracolumbar spine. The patient’s medical history revealed a congenital right foot deformity that had been surgically managed since the age of 13. Despite initial investigations of the lower extremities at age 17 that revealed scoliosis, a detailed examination at that time failed to identify multiple congenital abnormalities, which have now become apparent upon closer inspection (see Figure 1 for reference). Notably, the patient also had a longstanding diagnosis of a pilonidal cyst with recent symptoms of sub-febrile fever and persistent discharge, necessitating further investigation. It is noteworthy that the patient’s mother had an uncomplicated pregnancy and delivery, without a history of gestational diabetes, folate deficiency, maternal obesity, or exposure to teratogenic medications.

Neurological examination findings revealed intact cranial innervation. Weakness in dorsiflexion of the right foot (1–2 points) was noted, attributed to bone lengthening surgery performed at age 13, while muscle strength in other muscle groups was graded at 5 points. Romberg’s test demonstrated stability, and coordination tests were performed accurately. The patient exhibited an antalgic posture, and gait was cautious. A right-sided scoliotic curvature was observed, with bilateral equal tendon reflexes (+), and tenderness noted paravertebrally in the lumbar spine upon palpation. Meningeal symptoms were absent, and functions of the pelvic region remained unaffected.

**Figure 2:**
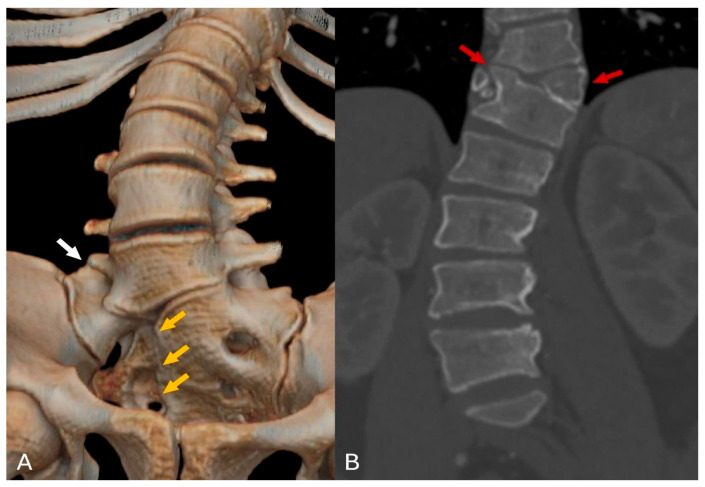
CT reconstruction and coronal CT imaging: (**A**) The 3D CT reconstruction reveals defects in the sacral vertebrae on the right side, indicative of type IVB hemisacrum (orange arrows), along with sacralization of the L5 vertebra on the right side, categorized as Castellvi classification IIIA (white arrow). (**B**) The coronal CT image displays a butterfly deformity at the Th11 level, accompanied by fusion of the Th11 and Th12 vertebrae on the right side (red arrows).

**Figure 3:**
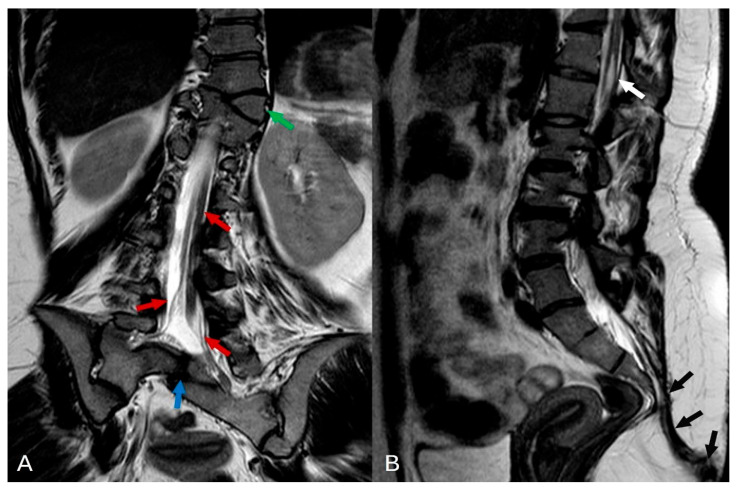
(**A**) In the T2-weighted turbo spin echo (TSE) coronal plane, several significant findings are noted: butterfly deformity in Th11 with right side of vertebrae fused to Th12 (green arrow), a split spinal cord with a single thecal sac indicative of diastematomyelia type II (red arrows), and partial segmentation of the sacral vertebrae (blue arrow). (**B**) In the T2 TSE sagittal plane, notable findings include a small syringohydromyelia (white arrow) observed at the thoracolumbar junction (Th12-L1 level), as well as the presence of a sinus pilonidal fistula (black arrows).

**Figure 4:**
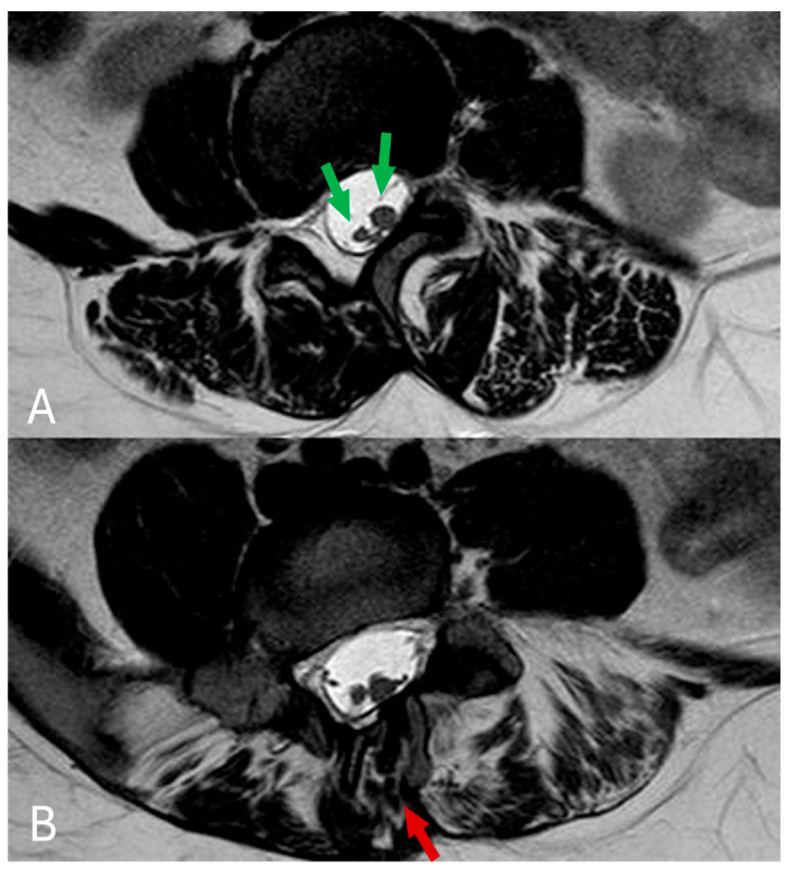
(**A**,**B**) In the axial plane, T2 TSE imaging reveals diastematomyelia across multiple levels (green arrows), along with evident defects in the vertebral posterior arch (red arrows).

The MRI examination of the thoracolumbar spine unveiled several intricate anatomical abnormalities. Scoliosis was evident within the thoracolumbar region, coupled with vertebral rotation. A butterfly deformity of the T11 vertebra was observed, with fusion of its right side to the T12 vertebral body. Multiple sacral vertebrae defects were identified, classified as type IVB hemisacrum according to Pang’s classification. Notably, a complete absence of the S5 and coccyx (Co1–5) vertebrae was confirmed, indicating full coccygeal agenesis. Further examination revealed sacralization of the L5 vertebra on the right side, classified as Castellvi IIIA. Posterior arch defects (spina bifida) were uniformly present across all sacral vertebrae (see Figure 2 for reference). Moreover, intricate spinal cord anomalies were noted, including conus medullaris positioned below the L2 level and diastematomyelia type II—characterized by a split spinal cord with a single thecal sac initiating from the L1 level. Remarkably, the dural sac extended seamlessly into the sacral region without any discernible inelastic structures. Additionally, a cystic collection indicative of syringomyelia was detected at the Th12-L1 level across T1, T2, and STIR sequences. Further pathology included multiple instances of disc protrusions and foraminal stenosis predominantly concentrated at the L4–L5 level. (see Figure 3 and Figure 4 for reference). Lastly, a sinus tract extending from the end of the spinal canal to the skin was identified. An additional pelvic MRI examination with an intravenous contrast agent was then performed at another hospital. The MRI demonstrated a tract with enhancing walls, which was initially interpreted as a pilonidal sinus. However, considering other spinal abnormalities and the absence of meningitis or spinal canal abscess, this finding could also be interpreted as a closed dorsal dermal sinus with inflammatory changes. Differentiating between the acquired (pilonidal sinus) and congenital (dorsal dermal sinus) nature of this finding was difficult, especially given the incomplete history of our patient. Histologic examination confirmed a pilonidal fistula with cyst and chronic purulent inflammatory changes.

The MRI findings align with characteristics typical of caudal regression syndrome (CRS), indicating the potential presence of additional anomalies warranting further investigation to comprehensively understand the anatomical changes. Subsequently, the patient was referred for specialist evaluation. A pelvic MRI confirmed a pilonidal sinus with inflammatory changes, necessitating consultation with a coloproctologist to determine the best surgical treatment approach. Additional diagnostic tests, including electrocardiography (ECG), echocardiography, CT of the thorax and abdomen (revealing an additional artery from the arteria iliaca communis dextra in the right kidney), nerve conduction study, thyroid ultrasonography (showing a TIRADS 3 nodule in the left lobe), head MRI, and gynecologic ultrasonography (identifying a left ovarian cyst and right para-tubal cyst), were performed, yielding normal or incidental findings.

Imaging and symptomatology did not indicate the need for neurosurgical spine intervention. While some radiologic signs of tethered spinal cord (low conus medullaris, diastematomyelia) were present, consultation with a neurosurgeon determined that the lower back pain was not directly linked to these findings. Tethered cord syndrome was ruled out. Clinical and radiologic findings confirmed a pilonidal sinus with inflammatory changes. Surgical excision was successful, and histologic examination of the postoperative material confirmed a pilonidal cyst with chronic purulent inflammation. The postoperative period was uneventful. Comprehensive multidisciplinary management and follow-up were recommended to address specific symptomatic and structural concerns arising from the intricate array of abnormalities observed.

## 3. Discussion

We describe a rare case of CRS in an adult. There are plenty of articles available describing this disease in pediatric patients; however, few adult cases are available in the literature. By further investigating different radiologic changes in adults we could better understand the degenerative changes that affect these patients in their lifetime and provide appropriate treatment options. Also, the provided diagnostic images showing changes in three different modalities could be useful for radiologists in general practice to better understand this disease and its variable anatomical deformities, improving diagnostic accuracy and patient clinical outcomes.

CRS is a congenital malformation that impacts the development of the lower spine and sacrum. While CRS is typically diagnosed during childhood due to its characteristic symptoms, documented cases in adulthood showcase a wide range of clinical features, emphasizing the variability and complexity of this condition in different age groups. Numerous studies have reported adult-onset CRS with diverse presentations, highlighting the importance of recognizing and understanding this condition beyond childhood (see Table 2 for comparison).

For example, Kumar et al. described a 30-year-old woman presenting with long-standing lower extremity weakness, paresthesia, urinary incontinence, and worsening back pain. MRI findings revealed abrupt termination of the spinal cord at T12, blunt-ending conus medullaris, and sacrococcygeal hypoplasia with absent S3–5 and coccygeal segments, alongside dysplastic S1 and S2 segments, no other pathologies were identified in visible parts of spinal cord [22].

Similarly, Yadav et al. reported a 20-year-old male with lifelong episodic involuntary leg movements. As recommended by neurologists, a MRI of the head was performed with no significant findings. Physical examination revealed associated left clubfoot. A spinal MRI revealed a low-lying spinal cord extending to S2, fused with a skin-covered, multiseptated cystic lesion through a posterior sacral defect. A native CT scan was also performed for better evaluation of bone structures. Additional findings included a deformed sacrum with fewer segments, absent coccyx, L5-S1 sagittal cleft, and a vertically split spinal cord with syrinx from Th12-L5 [23].

In another case, Bayraktutan et al. presented a 20-year-old woman with narrow hips, hypoplastic gluteal muscles, and a neurogenic bladder. MRI findings showed a hypoplastic sacrum, absent coccyx, rib fusion, diastematomyelia (split by midline osseous spur), and various vertebral anomalies such as hemivertebra, butterfly vertebra, and fused vertebrae leading to rotoscoliosis [24].

These cases highlight the significant variability in clinical presentation and radiographic findings associated with adult-onset CRS. While lower back pain, skeletal anomalies, and spinal cord abnormalities are consistent features, the severity and combination of neurological symptoms can vary widely among adult patients. As a result, individuals with milder or delayed symptoms may face misdiagnosis and inappropriate treatment for many years, as demonstrated in our case.

Tethered cord syndrome should be considered in CRS patients, especially with diastematomyelia or other spinal cord malformations or lesions. In the literature, tethered cord in adults typically presents with back pain and sphincter dysfunction, correlating with radiologic signs. While the described patient had lumbar pain associated with pilonidal sinus infection, no other clinical signs or sphincter dysfunction were present. Despite some radiologic signs of a fixed spinal cord in MRI examinations, consultations with neurologists and neurosurgeons determined that no further surgical interventions were necessary.

The complexity of CRS underscores the importance of thorough investigation and multidisciplinary evaluation when encountering adults with uncommon and nonspecific symptoms suggestive of spinal cord malformations. A collaborative approach involving neurologists, orthopedic surgeons, radiologists, and other specialists is essential for timely and accurate diagnosis, preventing unnecessary surgeries, and ultimately improving the quality of life for affected individuals. Early recognition and comprehensive management of adult-onset CRS are crucial for optimizing outcomes and addressing the multifaceted challenges associated with this rare congenital anomaly throughout the lifespan.

## 4. Conclusions

This report emphasizes the missed diagnosis of CRS in a 27-year-old woman with lower back pain. Despite some clear radiological signs ten years ago, CRS was not considered then. It stresses the need for healthcare professionals to stay alert to uncommon diagnoses, especially when initial tests do not provide clear answers.

This clinical case demonstrates a highly valuable and educative radiological finding. In the literature, such cases with radiological findings in adults are still lacking. We believe it will be interesting for readers and will be an additional contribution to science.

Early recognition of CRS and similar conditions is crucial for improving patient outcomes and quality of life through timely diagnosis. This enables tailored treatment strategies, including rehabilitation, orthopedic interventions, and urological management, which can prevent unnecessary procedures, reduce healthcare costs, and minimize the patient discomfort associated with delayed diagnosis and treatment. Emphasizing the significance of considering rare diagnoses like CRS in adults with atypical symptoms underscores the importance of thorough clinical evaluation, radiological interpretation, and interdisciplinary collaboration among healthcare providers to achieve accurate diagnoses and optimize patient care. Ongoing education and awareness are essential for enhancing the recognition and management of CRS and similar conditions in clinical practice.

## Figures and Tables

**Table 1:** Comparison of the sacral agenesis classifications.

Pang’s Classification [18]	Renshaw’s Classification [19]
Type I: Total sacral agenesis with missing lumbar vertebrae and ilia articulation where lower vertebral bodies or ilia are fused or articulating with the other side below the last vertebrae. Transverse pelvic diameter is severely shortened in the case of fused ilia.	Type I: Partial or complete unilateral sacral agenesis.
Type II: Total sacral agenesis with or without fused or articulating ilia, lumbar vertebrae are in place. Transverse pelvic diameter is severely shortened in the case of fused ilia.	Type II: Partial sacral agenesis with a partial but bilaterally symmetrical defect and a stable articulation between the ilia and a normal or hypoplastic first sacral vertebra
Type III: Subtotal sacral agenesis, at least S1 is present, sacrum lacks variable count of caudal segments, transverse pelvic diameter is normal.	Type III: Variable lumbar and total sacral agenesis with the ilia articulating with the sides of the lowest vertebrae present.
Type IV: Total or subtotal hemisacrum, missing variable count of vertebrae unilaterally or bilaterally.	Type IV: Total sacral and variable lumbar agenesis with the ilia fused or articulating below L5 level.
Type V: Total or subtotal coccygeal agenesis	Type V: Sirenomelia—a most severe form of sacral agenesis with variable limb deformities.

**Table 2:** Comparison of adult cases.

	Our Case	Case Described by Kumar et al. [22]	Case Described by Yadav et al. [23]	Case Described by Bayraktutan et al. [24]
**Clinical presentation**	One month of lower back pain, subfebrile fever.	Extremity weakness, paresthesia, urinary incontinence, back pain.	Episodic involuntary leg movements.	Narrow hips, hypoplastic gluteal muscles, and neurogenic bladder.
**Maternal diabetes**	No history of maternal diabetes.	Not mentioned.	Positive history of maternal diabetes.	Not mentioned.
**Available radiologic examinations**	X-ray, US, thoracic and abdominal CT, MRI of brain, thoracolumbar spine, pelvis.	MRI of lower back and pelvis.	MRI of brain, MRI and CT of lumbar spine and pelvis.	MRI of cervical and thoracolumbar spine.
**Radiologic findings in spinal column**	Th11-Th12 vertebral anomalies. Hemisacrum type IVB. Scoliosis with vertebral rotation. S5 and coccygeal agenesis, sacralization of L5.	Sacrococcygeal hypoplasia, including the absent S3–5 and coccygeal segments with dysplastic S1 and S2.	Deformed sacrum with S1–S3 segments, coccygeal agenesis. A sagittal cleft in L5 and S1 vertebral bodies.	Hypoplastic sacrum with preserved S1, coccygeal agenesis, vertebral anomalies-hemivertebra, butterfly vertebra, and fused vertebrae. Rotoscoliosis.
**Radiologic findings in spinal cord**	Conus medullaris below level of L2. Diastematomyelia type II. Syringomyelia at level Th12-L1.	High termination of spinal cord with a blunt-ending conus medullaris (club shaped), otherwise unremarkable.	Diastematomyelia type II. Syringomyelia in the right hemicord at Th12-L5 level.	Diastematomyelia type II with midline osseous spur.
**Other associated anomalies**	Congenital right foot deformity. Fused ribs.	Not mentioned.	Left club foot, multiseptated skin covered cystic lesion.	Fused ribs.
**Additional pathologies**	Pilonidal sinus. Additional right kidney artery. Benign thyroid nodule. Left ovarian cyst and para-tubal cyst.	Not mentioned.	Not mentioned.	Not mentioned.

## Data Availability

The data presented in this study are available on request from the corresponding author.
